# Global Transcriptomic Analysis Reveals Differentially Expressed Genes Involved in Embryogenic Callus Induction in Drumstick (*Moringa oleifera* Lam.)

**DOI:** 10.3390/ijms222212130

**Published:** 2021-11-09

**Authors:** Endian Yang, Mingyang Zheng, Xuan Zou, Xiaoling Huang, Heyue Yang, Xiaoyang Chen, Junjie Zhang

**Affiliations:** 1 Department of Forestry, College of Forestry and Landscape Architecture, South China Agricultural University, Guangzhou 510642, China; 2274893976@stu.scau.edu.cn (E.Y.); zmysndd@stu.scau.edu.cn (M.Z.); zxzouxuan@stu.scau.edu.cn (X.Z.); xlhuang12@scau.edu.cn (X.H.); yang15936509822@163.com (H.Y.); 2 Guangdong Key Laboratory for Innovative Development and Utilization of Forest Plant Germplasm, Guangzhou 510642, China; 3 Guangdong Province Research Center of Woody Forage Engineering Technology, Guangzhou 510642, China; 4 State Key Laboratory for Conservation and Utilization of Subtropical Agro-Bioresources, South China Agricultural University, Guangzhou 510642, China

**Keywords:** *Moringa oleifera*, embryogenic callus, transcriptome, differentially expressed genes

## Abstract

The plant embryogenic callus (EC) is an irregular embryogenic cell mass with strong regenerative ability that can be used for propagation and genetic transformation. However, difficulties with EC induction have hindered the breeding of drumstick, a tree with diverse potential commercial uses. In this study, three drumstick EC cDNA libraries were sequenced using an Illumina NovaSeq 6000 system. A total of 7191 differentially expressed genes (DEGs) for embryogenic callus development were identified, of which 2325 were mapped to the KEGG database, with the categories of plant hormone signal transduction and Plant-pathogen interaction being well-represented. The results obtained suggest that auxin and cytokinin metabolism and several embryogenesis-labeled genes are involved in embryogenic callus induction. Additionally, 589 transcription factors from 20 different families were differentially expressed during EC formation. The differential expression of 16 unigenes related to auxin signaling pathways was validated experimentally by quantitative real time PCR (qRT-PCR) using samples representing three sequential developmental stages of drumstick EC, supporting their apparent involvement in drumstick EC formation. Our study provides valuable information about the molecular mechanism of EC formation and has revealed new genes involved in this process.

## 1. Introduction

The plant embryogenic callus is an irregular embryogenic cell mass with strong regenerative ability. Under certain conditions, embryogenic callus cultured in vitro can regenerate into adventitious buds or form complete plants after somatic embryogenesis [1]. Although a callus can be generated at explant wound sites under the stimulation of exogenous hormones, only a small proportion of callus cells can differentiate into embryogenic callus. In addition to its frequent use for propagating various economically important species, embryogenic callus-based genetic transformation has emerged as the most successful plant transformation system in the last decade [2,3,4]. In plant tissue cultures, embryogenic callus formation is generally induced by optimizing the ratio of hormones in the medium [5,6,7]. Although hormones play central roles in embryogenic callus induction, this non-genetic method of optimizing culture conditions cannot provide culture schemes suitable for all genotypes [8,9]. Recent evidence indicates that embryogenic callus formation is a complex dynamic process that involves many intracellular metabolic changes and is influenced by various external environment factors [10,11].

*Moringa oleifera* Lam., commonly known as drumstick, belongs to the monogeneric family *Moringaceae* and is naturally distributed in the sub-Himalayan areas of India and various tropical African countries [12]. Drumstick is a tree with many potential commercial uses [13]. The vegetative organs have high levels of protein, vitamins, minerals, and phytochemicals and the seeds have high oil content [14]. In addition, this plant has a number of medicinal properties linked to antimicrobial [15], anti-inflammatory [16], detoxification [17], and anticancer activities [18]. Although it is a fast-growing tree species, it is difficult to quickly obtain good varieties through conventional breeding. Drumstick is dominated by outcrossing [19], the cultivated populations are separated in traits, and the seed activity declines quickly. It is difficult to obtain seedlings with consistent and uniform traits in production. Genetic engineering is an effective alternative method for introducing value-adding genes to modify important agronomic traits. Agrobacterium-mediated transformation via embryogenic callus has been used as a standard and reproducible method for transformation of many plants [20,21]. Embryogenic callus induction is therefore seen as a key step in transformation. We have developed an Agrobacterium-mediated transformation system for drumstick [22], but it has not been widely used due to its low transformation efficiency, which is largely due to the difficulty of inducing embryogenic callus formation. Embryogenic callus induction is thus a key bottleneck in drumstick regeneration and hinders its transgenic breeding. Ways of improving the rate of embryogenic callus induction are therefore needed to facilitate genetic engineering of this species.

Preliminary studies on drumstick embryogenic callus induction revealed that exogenous hormones had strong effects on induction, but the rates of induction and differentiation of embryogenic callus were low and genotype-dependent. Moreover, the changes in cellular structures and physiological status that occur during embryogenic callus induction are largely unknown, as are the details of the regulation of this process at the molecular level.

Callus induction is a complex biological process of cell dedifferentiation and redifferentiation that is regulated by genetic and epigenetic mechanisms [11,23]. Despite the importance of EC for drumstick genetic engineering, few studies have investigated the mechanisms underlying EC induction in this species. Transcriptome sequencing, assembly, and annotation is valuable because it allows large amounts of information to be gathered on transcribed regions relatively quickly and inexpensively. Therefore, to clarify the molecular mechanisms underlying acquisition of embryogenic potential during this process, a comparative analysis of transcriptome dynamics at different stages of EC induction was conducted. The major pathways and key events in embryogenic callus development were identified through deep analysis of differentially expressed genes (DEGs) together with histological and chemical analyses of samples representing different stages of embryogenic callus formation. This led to new insights into the molecular regulatory networks regulating embryogenic callus formation. Our results provide valuable information on the molecular mechanism by which somatic cells are converted into embryogenic callus in drumstick and reveal novel genes involved in this process.

## 2. Results

### 2.1. Morphological, Histological, and Biochemical Features

**Figure 1:**
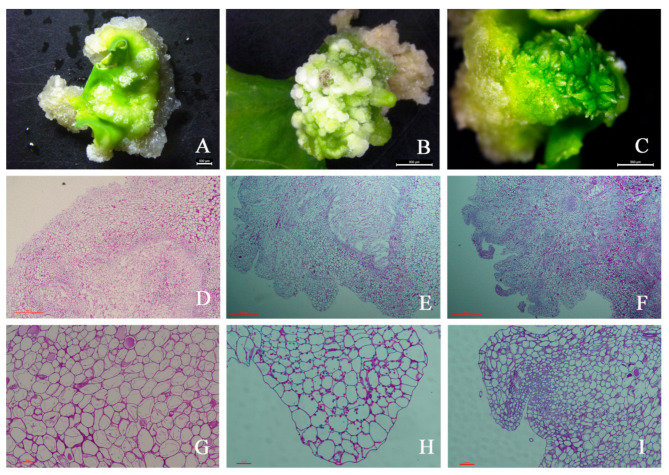
Morphological and histological features of different stages in the induction of drumstick embryonic callus during in vitro culture. (**A**–**C**) typical explant morphology on the 10th (**A**), 20th (**B**), and 30th (**C**) days of cultivation and (**D**–**I**) histological section of the callus on the 10th (**D**,**G**), 20th (**E**,**H**), and 30th (**F**,**I**) days of cultivation. Scale bars, (**A**–**C**)—500 μm, (**D**–**F**)—500 μm, (**G**–**I**)—50 μm.

The first morphological changes during the regeneration process occurred during the first ten days of culture, when the leaf explants became slightly swollen and intense cell proliferation was observed at the cut explants surface, giving rise to the callus (Figure 1A). Callus growth progressed and after ten days of culture, the first meristemoids were observed as small structures on the surface of the explant (Figure 1B). These structures continued developing, originating shoot buds (Figure 1C).

Callus cells appeared in the outer layer of the leaf explants. These cells were more variable in size and shape than their progenitors and had a large central vacuole (Figure 1D,G). Unlike the initially loosely arranged cells, the outermost cells were relatively small and exhibited a high capacity for division, enabling expansion of the outer layer. During this period, the cells were uniformly colored and arranged neatly and regularly without intercellular space. The callus continued to differentiate and formed the bud primordium (Figure 1E,H). The bud primordia cells continued to divide, resulting in a substantial increase in the number of cells and the formation of one or more buds (Figure 1F,I). Histological examinations were performed using light microscopy on the initial leaf explant and at two stages of callus of drumstick development by staining with the Periodic acid Schiff (PAS) reagent to view starch reserves. Starch granules were detected in the parenchymatous cells in stage 2 callus (Figure 1H), indicating that embryogenic cells display metabolic competence.

### 2.2. Global Transcriptomic Changes at Different Stages of Embryogenic Callus Induction Revealed by Transcriptome Sequencing Analysis

To obtain a comprehensive overview of the drumstick transcriptome, RNA was extracted from the three different explants. The Illumina NovaSeq 6000 platform generated 44,952,482 clean reads containing a total of 57.76 Gb clean data with 94.86% Q30 bases (base quality > 30) after performing stringent quality assessment and data filtering. The quality of the sequencing data is summarized in Table 1. The clean reads were mapped to the drumstick genome using the HISAT2 tool. The average mapping ratio ranged from 93.33 to 95.58%. StringTie was used to perform transcript assembly based on the read alignments. After optimal gene structure prediction and alternative splicing analysis, 23,591 genes were identified including 4126 new genes.

To validate and annotate the assembled transcriptome library, we searched against the non-redundant (Nr) peptide database (ftp://ftp.ncbi.nih.gov/blast/db) (14 January 2021), the Swiss-Prot protein database (http://www.uniprot.org) (14 January 2021), KOG (http://www.ncbi.nlm.nih.gov/KOG) (14 January 2021), and KEGG (https://www.genome.jp/kegg) (14 January 2021) using BLASTx (http://blast.ncbi.nlm.nih.gov/Blast.cgi) (14 January 2021) with a cutoff E-value of 10^−5^. It was found that 90.88% of the transcripts had significant similarity to at least one target from these databases (Table 2).

### 2.3. Functional Annotation and Classification of Genes Related to Embryogenic Callus Induction

**Figure 2:**
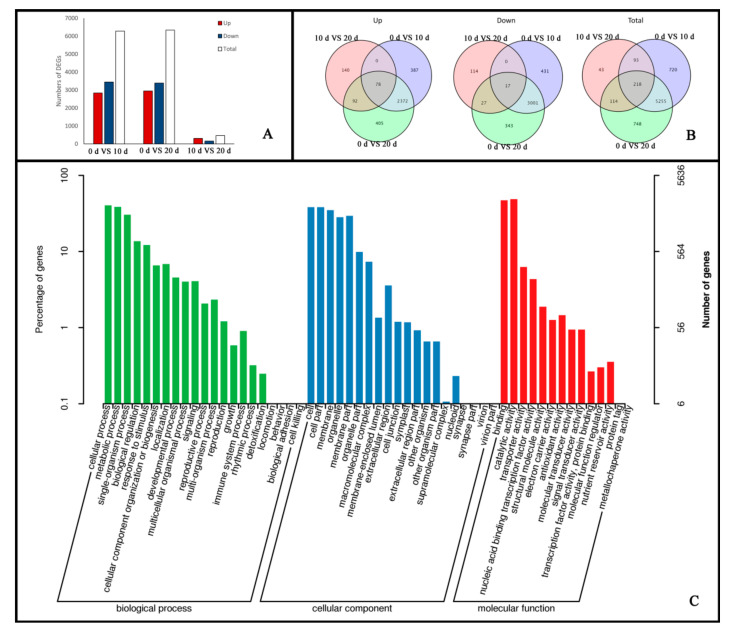
(**A**) Functional annotation and classification of genes related to embryogenic callus induction. Numbers of up- and down-regulated DEGs in different stages of embryogenic callus induction; (**B**) Venn diagram of annotated transcripts in multiple databases; (**C**) GO classification of DEGs in embryogenic callus induction.

DEGs were classified as embryogenic callus induction responsive genes if they exhibited a Fold Change ≥ 2 and FDR < 0.01 at any development point when compared to the control according to DESeq2. In total, 7191 DEGs were identified during embryogenic callus development. Of these DEGs, 6286 were differentially expressed between zero-day and ten-day explants, with 2837 being upregulated and 3449 downregulated. Only 468 DEGs were identified from ten-day explants to twenty days, of which 310 DEGs were upregulated and 158 DEGs were downregulated (Figure 2A). Additionally, 5975 DEGs were only differentially expressed in ten-day explants and 157 were only differentially expressed in twenty-day explants; 78 genes showed increased expression in all developmental stages and 17 showed reduced expression in all stages (Figure 2B). These results suggest that there are significant differences in gene expression at different stages of embryogenic callus development and that many genes are needed to ensure complete differentiation.

To functionally classify the identified genes related to embryogenic callus induction, the DEGs were matched to the Gene Ontology (GO) database (http://www.geneontology.org) (14 January 2021). Three categories were represented: biological processes, cellular components, and molecular functions. Within these main GO categories, cellular process, cell, and catalytic activity had the greatest number of matches among the DEGs (Figure 2C).

**Figure 3:**
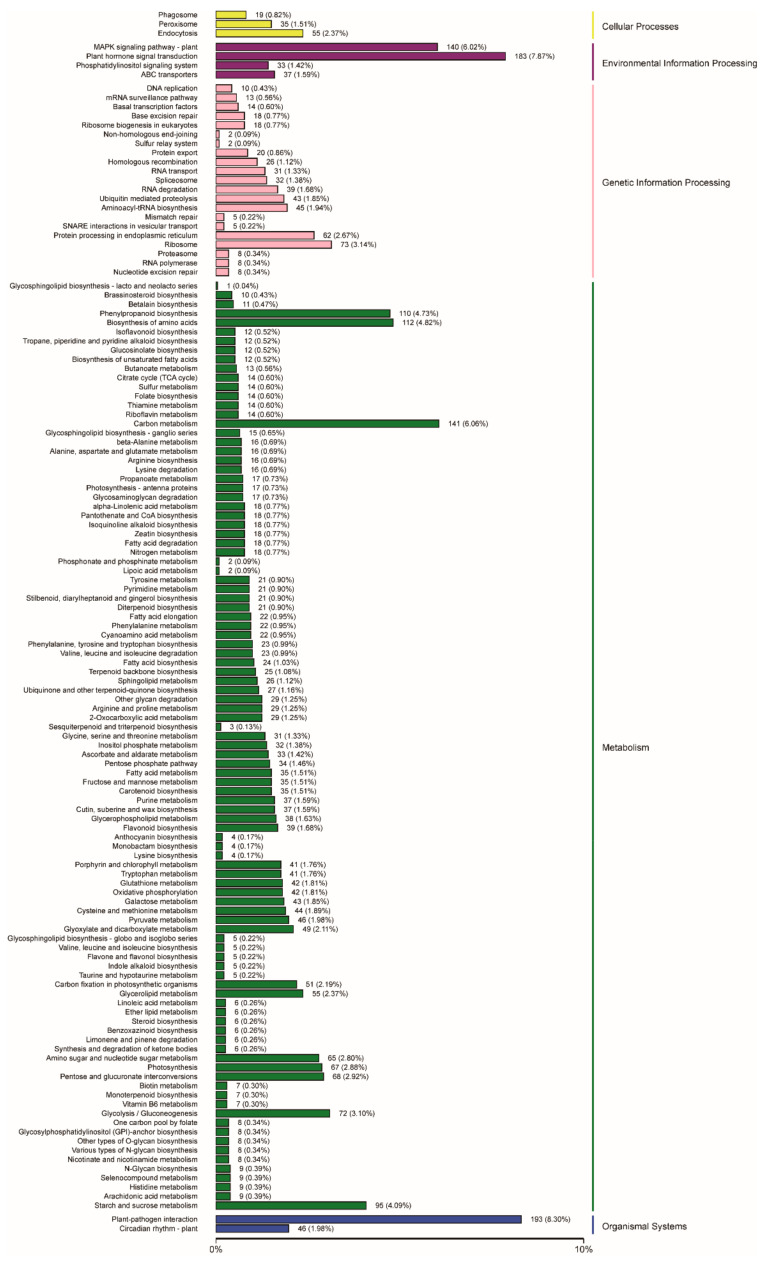
Functional classification and pathway assignment of DEGs by KEGG.

**Figure 4:**
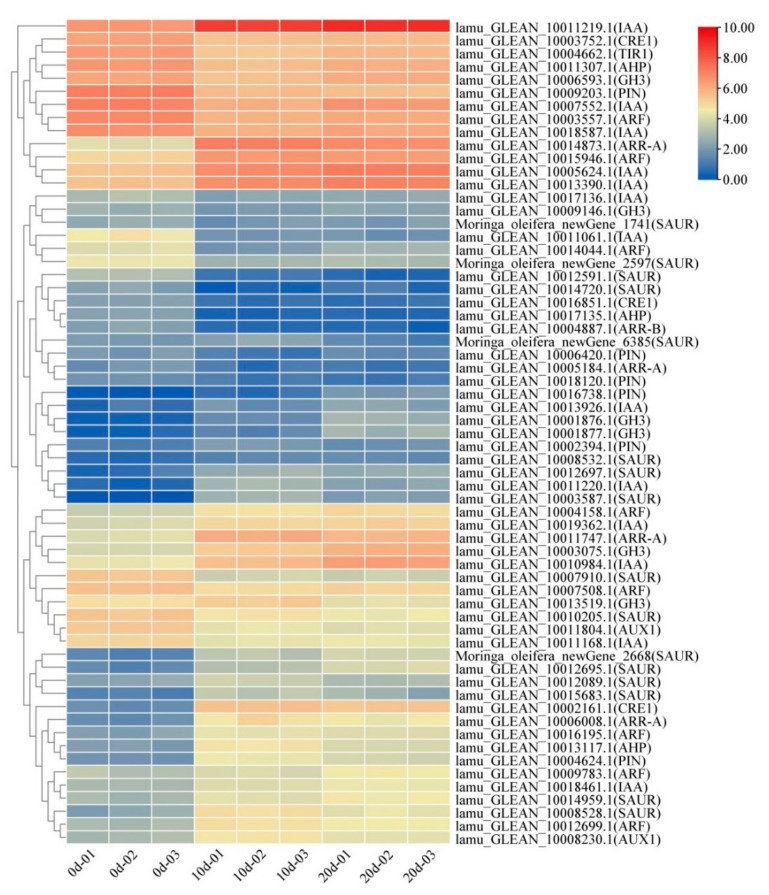
Heatmap of differentially expressed genes related to auxin and cytokinin metabolism. Columns and rows in the heatmap represent samples and genes, respectively. Samples’ names are shown below the heatmaps. The color bar explains the scale used to indicate the genes’ expression levels. The scale represents the logarithm of FPKM value of gene expression.

To identify metabolic pathways potentially related to embryogenic callus formation, the DEGs were analyzed using the KEGG (https://www.genome.jp/kegg) (14 January 2021) pathway analysis tool—a compilation of manually verified pathway maps used to categorize gene functions, with an emphasis on biochemical pathways. A total of 2325 (32.33%) DEGs were mapped to the KEGG database, with significant enrichment in the “metabolism” and “genetic information processing” clusters. In the “environmental information processing” category, “plant hormone signal transduction” genes were most highly represented among the DEGs, while “plant-pathogen interaction” genes were most highly represented in the “organismal systems” category (Figure 3 and Figure 4).

### 2.4. Auxin and Cytokinin Metabolism Involved in Embryogenic Callus Induction

Auxin is a key hormone that is included in EC induction media and must be maintained in balance with cytokinins during EC development. Fifty-one genes (2 *Aux*/*Lax* genes, 13 *Aux*/*IAA* genes, 8 *ARF* genes, 6 *PIN* genes, 6 *GH3* genes, and 16 *SAUR* genes) related to auxin signaling pathways were differentially expressed during embryogenic callus formation (Figure 4). Among them, one *Aux*/*Lax* gene, four *Aux*/*IAA* genes, three *ARF* genes, two *PIN* genes, three *GH3* genes, and eight *SAUR* genes were significantly up-regulated when comparing leaf explants to ten-day callus samples, and most of them remained highly expression in twenty-day callus samples. Changes in the expression of auxin signaling pathway related genes were visualized using a heatmap and those with similar expression patterns were categorized. Members of the same gene families showed different expression patterns, suggesting that the regulation of auxin signaling networks during callus differentiation is very complex. It is worth noting that the *IAA* gene (*LAMU_GLEAN_10011219.1*) was significantly up-regulated during callus formation and remained strongly expressed during callus differentiation, suggesting that it may be a key regulatory factor in the initiation and maintenance of callus regeneration. Further experimental studies are needed to confirm the function of this gene.

Because cytokinin plays a major role in EC induction, the expression patterns of genes encoding proteins involved in cytokinin signaling were also investigated. Eleven genes (four Type *A-ARR* genes, three *CRE1* genes, three *AHP* genes, and one Type *B-ARR* genes) related to cytokinin signaling pathways were differentially expressed. Cytokinins rapidly activate the expression of type-*A ARRs*, a family of genes encoding proteins thought to act as feedback repressors of cytokinin responses. Four type *A-ARR* genes were differentially expressed, and three were upregulated significantly during EC formation. One of these genes, *LAMU_GLEAN_10014873.1*, was strongly expressed at both stages of callus development, being upregulated by more than a factor of seven.

### 2.5. Embryogenesis-Labeled Genes Involved in Embryogenic Callus Induction

**Figure 5:**
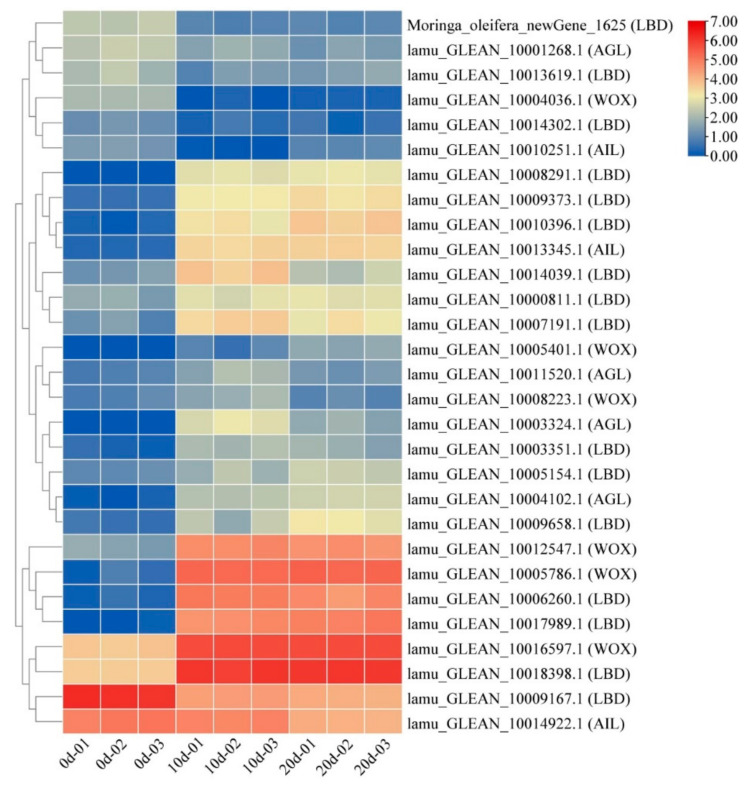
Heatmap of embryogenesis-labeled genes. Columns and rows in the heatmap represent samples and genes, respectively. Samples names are shown below the heatmaps. The color bar explains the scale used to indicate the genes’ expression levels. The scale represents the logarithm of FPKM value of gene expression.

In addition to plant hormone signaling pathway genes, several embryogenesis-labeled genes have been reported to play roles in embryogenic callus induction. To characterize the embryogenesis-labeled genes during drumstick EC induction, a comparative analysis of expression levels at different stages of induction was performed. Twenty-nine differentially expressed embryogenesis-labeled genes were found, belonging to the *LBD*, *AGL*, *WOX*, and *AIL* gene families (Figure 5). Most of these DEGs such as *WOX* (*LAMU_GLEAN_10016597.1*) and *LBD* (*LAMU_GLEAN_10019398.1*) were up-regulated at the callus initiation stage and remained highly expressed at the later stage, indicating that these genes were not only involved in EC induction, but also played important roles in maintaining the cells’ embryonic state.

### 2.6. Transcription Factors Involved in Embryogenic Callus Induction

**Figure 6:**
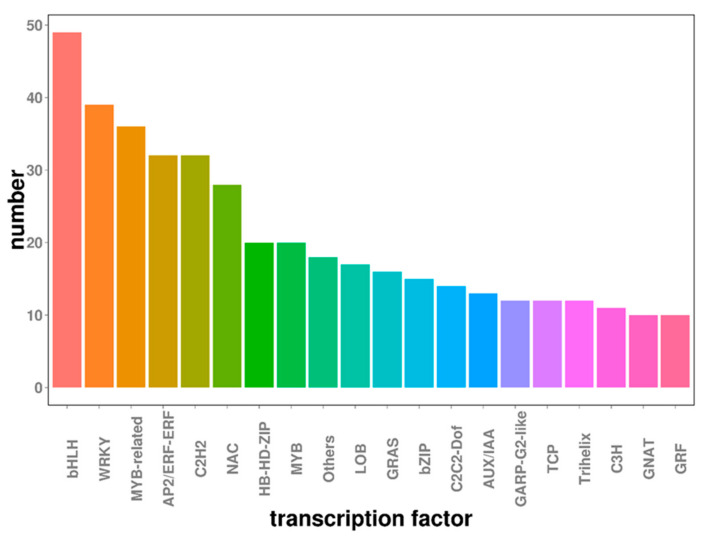
Transcription factors involved in embryogenic callus induction.

Transcription factors are proteins that bind specifically to a particular gene sequence and enable spatiotemporal control over the target gene’s expression. Previous studies confirmed that transcription factors play important roles in embryogenic callus formation. There were 589 transcription factors from 20 different families among the DEGs identified in this study (Figure 6). The three transcription factor families most heavily represented among this group were the *bHLH*, *WRKY*, and *MYB*-related families, which collectively accounted for 21% of all differentially expressed transcription factors. Previous studies showed that these gene families were differentially expressed under stress conditions and served to link stress to developmental pathways. All major TF families were represented in the mRNA transcriptome at each developmental stage and several TF families were specifically expressed during particular developmental periods.

### 2.7. Expression Patterns of Selected DEGs

To experimentally confirm the expression of the unigenes identified by sequencing and computational analysis, 16 representative DEGs related to auxin signaling pathways including *Aux*/*IAA*, *SAUR*, *ARF,* and *PIN* were selected for qRT-PCR analysis across the three sequential developmental stages of drumstick EC.

**Figure 7:**
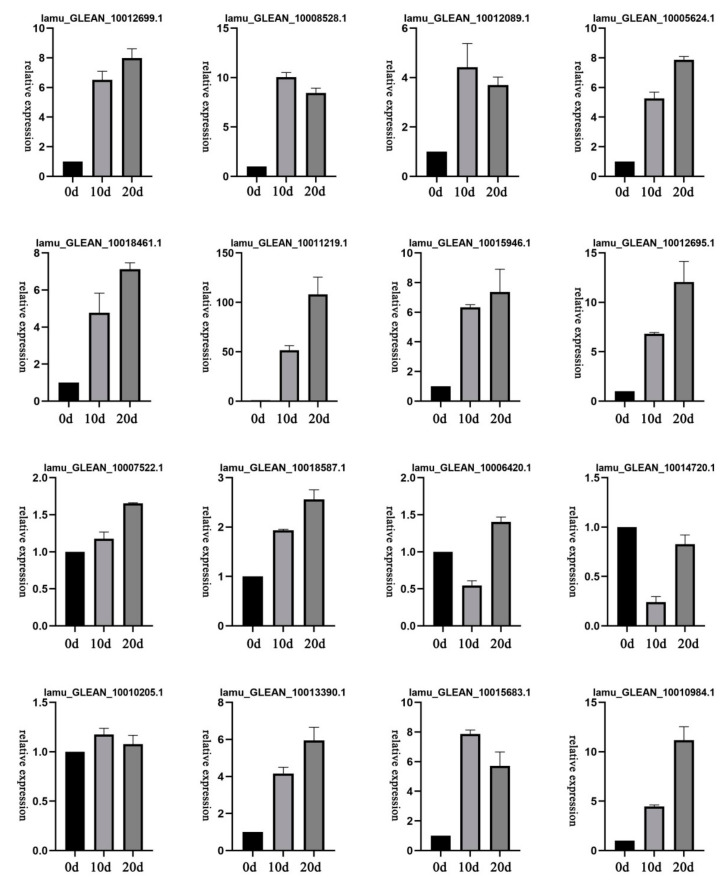
Expression level validation by qRT-PCR for selected genes in three stages.

Based on the analyzed qRT-PCR data (S1, Figure 7), all of these DEGs were expressed at varying levels in different stages. Moreover, the qRT-PCR results were generally consistent with the RNA-seq data, indicating the reliability of the transcriptome analysis. These results also suggest that all of the tested genes may play important roles in auxin transport and contribute to asymmetric auxin distributions that lead to EC induction.

## 3. Discussion

### 3.1. Auxin and Cytokinin Play an Important Role in Drumstick EC Formation

Auxin and cytokinins play key roles in plant cell division and differentiation [9]. It is known that auxin activates the expression of three major families of primary response genes: the *Aux*/*IAA* genes, the *GH3* genes, and the *SAUR* genes [24]. *Aux*/*IAA* family members are short-lived nuclear proteins that for homo- and heterodimers with other *Aux*/*IAA* and *ARF* genes to suppress transcription of downstream auxin-responsive genes [25]. In our study, 13 *Aux*/*IAA* genes were differently expressed during EC formation, while 5 *Aux*/*IAA* genes were significantly up-regulated in ten-day callus and remained strongly expressed in twenty-day callus. The expression level of one *Aux*/*IAA* family member was significantly higher than that of other genes in the pathway, indicating that this gene may play an important role in callus induction. The *SAUR* family is the largest known family of early auxin-inducible genes [26], and *OsSAUR39* was reported to negatively regulate auxin biosynthesis and transport [27]. Interestingly, several *SAUR* genes were differentially expressed in the early stage of drumstick callus induction and showed different expression patterns. Consequently, their roles in this process need further study. *ARF* transcription factors are key components of the auxin signal transduction pathway that inhibit or activate the expression of downstream genes by binding to upstream response elements of auxin response genes [28]. Four *ARF* genes were up-regulated during EC induction, suggesting these genes may play important roles in callus development.

Cytokinins regulate plant development via classic two-component regulatory systems [24]. Previous studies have shown that *A-ARR* genes are up-regulated under cytokinin stimulus, which would repress *WUS* expression to maintain normal shoot apical development. Moreover, *CRE1* is an upstream regulator of *A-ARR* together with *AHP* and *B-ARR* [29]. One *CRE1* gene and 3 *A-ARR* genes were significantly up-regulated during EC induction, suggesting that they play important roles in cytokinin signaling during this process. These results will provide a foundation for further studies on the effects of cytokinin signaling in drumstick.

### 3.2. Embryogenesis-Labeled Genes Play Important Roles in Drumstick EC Formation

Several genes have been identified as molecular markers of SE, including somatic embryogenesis receptor-like kinase (*SERK*), leafy cotyledon 1 (*LEC1*), BABYBOOM (*BBM*), wuschel (*WUS*), and WUS-related homeobox (*WOX*) [30]. *SERK* is known to play a key role in the acquisition of embryogenic competence in plant cells [31]. However, no genes of this family were differentially expressed during the developmental stages examined in this work. Conversely, *LBD*, *AGL*, *WOX*, and *AIL* genes were differentially expressed in different induction stages. Some *LBD* genes have been identified as key regulators of callus formation in tissue culture. For example, *LBDs* reportedly interact physically with the *AtbZIP59* transcription factor to mediate auxin-induced callus formation in *Arabidopsis* [32]. *AGL15* is a MDAS-BOX protein that is preferentially expressed in developing embryos and promotes somatic embryo (SE) initiation [33]. *WOX* genes were markers of cell fate during early embryogenesis in *Arabidopsis* [34], and *WOX2* and *WOX9* were highly expressed in the early stage of SE development in *Picea abies*, suggesting that these genes may function together during conifer embryo patterning [35]. *WOX4* is associated with callus proliferation, and *WOX1* is involved in root primordium initiation in the shrub species *Jasminum sambac* [36].

### 3.3. Stress-Related Transcription Factors Play an Important Role in Drumstick EC Formation

Stress is an important regulatory signal for cell dedifferentiation that can induce the expression of many transcription factors, cell fate reorganization, and embryogenic acquisition [37]. It is clear that excising tissue from a parent plant, cutting it into pieces, and putting into an artificial culture environment imposes considerable stress on the plant tissue. The connection between stress and EC initiation has received increasing attention in recent years.

In this study, several transcription factors exhibited dynamic changes in expression during different developmental stages of EC formation; examples include *bHLH*, *WRKY*, the *MYB*-related family, and *AP2/ERF*. Previous studies have shown that these transcription factors play important roles in responding to various stresses [38,39]—they are differentially expressed under stress conditions and act to link stress to developmental pathways. *bHLH* participates in the process of adaptation to various stresses and also has pleiotropic effects during cell proliferation and differentiation [38]. In this study, 19 *bHLH* members were upregulated and 17 were downregulated when comparing leaf explants to ten-day callus tissue. Conversely, when comparing ten and twenty-day callus samples, two *bHLH* members were upregulated and only one was downregulated. These results indicated that more *bHLH* transcription factors are needed to initiate differentiation in the early stage of callus differentiation. *WRKY* is involved in responses to biotic and abiotic stresses and hormone signaling [39]. *WRKY* transcription factors exhibited complex expression patterns during drumstick callus induction, suggesting that these genes may perform different regulatory functions. *MYB*-related is involved in hormone and environmental responses [40]. Previous studies have confirmed that the *AP2/ERF* transcription factor plays an important role in callus induction and adventitious bud regeneration. *WIND1* is an *AP2/ERF* transcription factor that was shown to promote *Arabidopsis* shoot regeneration [41]. In this study, we identified 40 differentially expressed *AP2/ERF* transcription factors, most of which were upregulated during EC initiation. It is notable that the *ARF*, *Aux/IAA* and *LBD* families also have roles in auxin-mediated signaling [42]. Further study of these genes is expected to reveal ways of improving the reliability and efficiency of embryogenic callus induction in drumstick.

## 4. Material and Methods

### 4.1. Induction of Embryogenic Callus and Plant Regeneration

The donor plants used in this study were in vitro-grown shoots of clonal drumstick tree M-2 (South China Agricultural University). Stage 2 leaves from M-2 were wounded and precultured on Murashige and Skoog (MS) basal medium supplemented with 0.8 mg/L 6-benzyladenine (BA), 0.2 mg/L kinetin (KT), and 0.05 mg/L 1-naphthaleneacetic acid (NAA) to heal the cutting wound and initiate cell division. All media used were adjusted to pH 5.8–6.0 with 1 *N* NaOH or 1 *N* HCl solution, set using 0.6% (*w*/*v*) agar, 30 g/L sucrose and autoclaved at 121 °C for 20 min. All cultures were kept under cool white light (about 50 µmol m^−2^ s^−1^) with a 12 h photoperiod and a temperature of 25–27 °C.

### 4.2. Histomorphological and Histochemical Analyses

Fresh explants were observed under an Olympus SZX10 (Olympus, Guangzhou, China) stereomicroscope after preparation for microscopic evaluation. Explants for light microscopic examination were dissected and fixed in glutaraldehyde, dehydrated in a series of ethanol solutions, then infiltrated and embedded in paraffin. Sections (thickness: 5 μm) were cut using a Leica RM2235 (Leica, Shanghai, China) rotary microtome. Periodic acid-Schiff (PAS) was used to detect insoluble polysaccharides.

### 4.3. Library Preparation and Transcriptome Sequencing

Drumstick leaf explants cultured for ten and twenty days exhibited clearly distinct morphology, so three sets of explants were collected at each of the three sequential developmental stages (0, 10, and 20 d). For RNA seq analysis, fresh samples were collected, immediately immersed in liquid nitrogen, and then stored at −80 °C. RNA was extracted using the Simple Total RNA Kit (OMEGA, Guangzhou, China) according to the manufacturer’s instructions. RNA concentration and purity were measured using a NanoDrop 2000 system (Thermo Fisher Scientific, Wilmington, DE, USA). RNA integrity was assessed using the RNA Nano 6000 Assay Kit of the Agilent Bioanalyzer 2100 system (Agilent Technologies, CA, USA). Afterwards, 1 µg RNA of per sample was employed for the RNA-seq analysis using Illumina NovaSeq 6000 system by Biomarker Technologies (Beijing, China). The maximum read length of this system is 2 × 250 bp. Sequencing libraries were generated using the NEBNext Ultra^TM^ RNA Library Prep Kit for Illumina (NEB, Ipswich, MA, USA). Clustering of the index-coded samples was performed on a cBot Cluster Generation System using the TruSeq PE Cluster Kit v.4-cBotHS (Illumina). Libraries were sequenced on an Illumina platform and paired-end reads were generated. HISAT2 v2.0.5 (http://ccb.jhu.edu/software/hisat2/index.shtml) (4 November 2016) tools software was used to map the sequences to the reference genome, which has been deposited in National Center for Biotechnology Information database (NCBI) under the accession number PRJNA268707. Gene function was annotated from commonly used databases. DEGs were observed using DESeq2 software and parameters were a false discovery rate (FDR) ≤ 0.01 and |log2FC| (FC, fold change) ≥ 1. Finally, RNA-seq data in this study had been submitted to https://www.ncbi.nlm.nih.gov/ (4 November 2016) and the accession number was PRJNA771463.

### 4.4. Real-Time Quantitative PCR

Sixteen genes with potential roles in drumstick embryogenic callus were chosen for validation using real-time quantitative PCR (RT-qPCR) in three cultured stages that were consistent with transcriptome sequencing. The cDNA first-strand was synthesized from total RNA using First-Strand cDNA Synthesis Kits and oligo-dT primers (Takara, Guangzhou, China). RT-qPCR was performed, and *ACP2* was amplified as a reference gene [43]. The forward and reverse primers listed in Table 3 were designed using Primer 5.0 and blasted in reference genome to verify that primers cover adjacent exons. Three technical replicates were performed for the test and reference genes of each sample to obtain precise and reproducible results. Relative expression levels for each gene were estimated using the 2^−ΔΔCT^ method. Statistical analysis was carried out using SPSS 22.0 software (SPSS Inc., Chicago, IL, USA), Duncan’s multiple range test was used to detect differences between means; *p*-values < 0.05 were considered significant.

## Figures and Tables

**Table 1:** Summary of sequence assembly after Illumina sequencing.

Samples	Clean Reads	Mapped Reads	Q20 (%)	Q30 (%)	GC (%)
0 d-1	44,952,482	42,277,829 (94.05%)	98.3	95.04	45.62
0 d-2	38,824,772	36,488,916 (93.98%)	98.24	94.98	45.56
0 d-3	43,843,258	41,495,852 (94.65%)	98.31	95.04	45.55
10 d-1	43,791,194	41,857,113 (95.58%)	98.31	95.08	45.05
10 d-2	43,102,782	40,697,612 (94.42%)	98.22	94.86	44.92
10 d-3	42,518,186	40,566,753 (95.41%)	98.25	94.92	45.01
20 d-1	41,091,584	38,900,779 (94.67%)	98.2	94.94	45.14
20 d-2	43,883,936	40,957,664 (93.33%)	98.21	94.93	45.31
20 d-3	44,517,654	41,830,480 (93.96%)	98.25	95.02	45.17

**Table 2:** Gene functional annotation results.

Annotated Databases	Annotated_Number	Percentage (%)	300 ≤ Length < 1000	Length ≥ 1000
COG	6337	26.86	1905	4359
GO	16,918	71.71	6809	9618
KEGG	13,097	55.52	4901	7915
KOG	11,384	48.26	4096	7034
Pfam	16,457	69.76	6321	9809
Swiss-Prot	15,255	64.66	5858	9011
eggNOG	2579	10.93	1156	1266
NR	21,382	90.64	9422	11,256
At least one database	21,439	90.88	9454	11,264

**Table 3:** Primer sequences for qRT-PCR analysis.

Gene ID	Forward Primers	Reversed Primers
ACP2.	GAAACCAATGAGCACCCAGC	GATGAATACCAGTCCACCGCAAC
lamu_GLEAN_10018587.1	ATCTCCTCCCTGCTGCTTG	CTTGACTTGTCGCCCTCTT
lamu_GLEAN_10010205.1	GGAGCAAACGCCACTGTCA	TTCATCACCCTCGCCATCA
lamu_GLEAN_10012089.1	CACCTGATTCCCTTCCATA	ATACTCTTCCTCGGCTTCC
lamu_GLEAN_10007552.1	GCACCCTTCTTCCTTCTG	TCCTCGGAGCTTGTCTTT
lamu_GLEAN_10006420.1	TGACAAGGATTTGGAGGGT	CAAGCGTAGGAGTTGGGAT
lamu_GLEAN_10008528.1	AGGAGGAAGGCCAGGATTAC	AAGATTGGGTGGTTGAGGTG
lamu_GLEAN_10012699.1	GCCCTCTTGTTTCTTTGCC	GTGATGGAAGATTCGGGTAGT
lamu_GLEAN_10012695.1	ATGGCGATCAGGAAGTCA	TTGCTCGTCGTGGTAGGTC
lamu_GLEAN_10010984.1	CACCAGACAGGGACGATG	ACCGCCACCACTACAACC
lamu_GLEAN_10018461.1	TGGAGGCTACTGAGGCTAGT	TGACAGACCACAGAAGGGA
lamu_GLEAN_10011219.1	GCTCTACGTGGGACGCTA	CACCTTCAGGCATTTACCG
lamu_GLEAN_10015946.1	GCTTACCTCCACAACTTATC	TAGGCATCCTTCTGCTCTT
lamu_GLEAN_10013390.1	CTACAGAACTGAGGCTTGG	AAGGAGTGGAAATAGTGGC
lamu_GLEAN_10015683.1	GAAGCCGAGTCCGAGTTTG	CCTTGACGAATACGACCACC
lamu_GLEAN_10014720.1	CTGCTGCTGTCAACAAGGT	GCCGCATCCAGTAATCTCA
lamu_GLEAN_10005624.1	GGGCTTGCCTGTTATGGT	ACTCAGTTGCTGGCTTTG

## Data Availability

All the raw data of the transcriptome are deposited in the NCBI under the accession number PRJNA771463.

## Supplementary Materials

The following are available online at https://www.mdpi.com/article/10.3390/ijms222212130/s1.

## References

[1] Zimmerman J.L. (1993). Somatic embryogenesis: A model for early development in higher plants. Plant Cell.

[2] Al Abdallat A.M., Sawwan J.S., Al Zoubi B. (2011). Agrobacterium tumefaciens-mediated transformation of callus cells of Crataegus aronia. Plant Cell Tissue Organ Cult..

[3] Elegba W., McCallum E., Gruissem W. (2021). Efficient genetic transformation and regeneration of a farmer-preferred Cassava cultivar from Ghana. Front. Plant Sci..

[4] Saika H., Toki S. (2010). Mature seed-derived callus of the model indica rice variety Kasalath is highly competent in Agrobacterium-mediated transformation. Plant Cell Rep..

[5] Li J., Zhang D., Que Q., Chen X., Ouyang K. (2019). Plant regeneration and Agrobacterium-mediated transformation of the miracle tree *Neolamarckia cadamba*. Ind. Crop. Prod..

[6] Passamani L.Z., Reis R.S., Vale E.M., Sousa K.R., Aragao V.P.M., Santa-Catarina C., Silveira V. (2019). Long-term culture with 2,4-dichlorophenoxyacetic acid affects embryogenic competence in sugarcane callus via changes in starch, polyamine and protein profiles. Plant Cell Tissue Organ Cult..

[7] Wang Y., Ruemmele B.A., Chandlee J.M., Sullivan W.M., Knapp J.E., Kausch A.P. (2002). Embryogenic callus induction and plant regeneration media for bentgrasses and annual bluegrass. In Vitro Cell. Dev. Biol.-Plant.

[8] Jabeen N., Chaudhry Z., Mirza B., Rashid H. (2005). Effect of genotype and explant type on in vitro shoot regeneration of tomato (*Lycopersicon esculentum* Mill.). Pak. J. Bot..

[9] Kadhimi A.A., Alhasnawi A.N., Isahak A., Ashraf M.F., Mohamad A., Doni F., Yusoffi W.M.W. (2014). Effect of Genotype and Growth Regulators in Induction of Embryogenic Callus in Rice. Pure Appl. Microbiol..

[10] Kang H., Lee C., Kwon S., Park J., Kang K., Shim D. (2021). Comparative transcriptome analysis during developmental stages of direct somatic embryogenesis in Tilia amurensis Rupr. Sci. Rep..

[11] Lai Z., Lin Y. (2013). Analysis of the global transcriptome of longan (*Dimocarpus longan* Lour.) embryogenic callus using Illumina paired-end sequencing. BMC Genom..

[12] Zhang J., Yang Y., Lin M., Li S., Tang Y., Chen H., Chen X. (2017). An efficient micropropagation protocol for direct organogenesis from leaf explants of an economically valuable plant, drumstick (*Moringa oleifera* Lam.). Ind. Crop. Prod..

[13] Lin M., Zhang J., Chen X. (2018). Bioactive flavonoids in Moringa oleifera and their health-promoting properties. J. Funct. Foods.

[14] Leone A., Spada A., Battezzati A., Schiraldi A., Aristil J., Bertoli S. (2015). Cultivation, genetic, ethnopharmacology, phytochemistry and pharmacology of Moringa oleifera leaves: An overview. Int. J. Mol. Sci..

[15] Sengupta M.E., Keraita B., Olsen A., Boateng O.K., Thamsborg S.M., Pálsdóttir G.R., Dalsgaard A. (2012). Use of Moringa oleifera seed extracts to reduce helminth egg numbers and turbidity in irrigation water. Water Res..

[16] Bakre A.G., Aderibigbe A.O., Ademowo O.G. (2013). Studies on neuropharmacological profile of ethanol extract of Moringa oleifera leaves in mice. J. Ethnopharmacol..

[17] Kituyi J.L., Foulkes M., Worsfold P., Ongulu R.A., Kiplagat A., Gachanja A. (2013). Efficiency of pre-treated Moringa oleifera for the removal of Cd^2+^ and Zn^2+^ ions from waste waters. Ecohydrol. Hydrobiol..

[18] Sreelatha S., Jeyachitra A., Padma P.R. (2011). Antiproliferation and induction of apoptosis by Moringa oleifera leaf extract on human cancer cells. Food Chem. Toxicol..

[19] Muhl Q.E., du Toit E.S., Steyn J.M., Apostolides Z. (2013). Bud development, flowering and fruit set of *Moringa oleifera* Lam. (Horseradish Tree) as affected by various irrigation levels. J. Agric. Rural. Dev. Trop. Subtrop..

[20] Batra S., Kumar S. (2003). Agrobacterium-mediated transient GUS gene expression in buffel grass (*Cenchrus ciliaris* L.). J. Appl. Genet..

[21] Takamizo T., Sato H. (2020). Protocol for Agrobacterium-mediated transformation of tall fescue and future perspective on the application of genome editing. Plant Biotechnol..

[22] Zhang J., Lin M., Chen H., Chen X. (2017). Agrobacterium tumefaciens-mediated transformation of drumstick (*Moringa oleifera* Lam.). Biotechnol. Biotechnol. Equip..

[23] Ikeuchi M., Sugimoto K., Iwase A. (2013). Plant callus: Mechanisms of induction and repression. Plant Cell.

[24] Che P., Gingerich D.J., Lall S., Howell S.H. (2002). Global and hormone-induced gene expression changes during shoot development in Arabidopsis. Plant Cell.

[25] Chen L., Zheng X., Guo X., Cui Y., Yang H. (2019). The roles of Aux/IAA gene family in development of Dendrocalamus sinicus (Poaceae: Bambusoideae) inferred by comprehensive analysis and expression profiling. Mol. Biol. Rep..

[26] Ren H., Gray W.M. (2015). SAUR proteins as effectors of hormonal and environmental signals in plant growth. Mol. Plant.

[27] Kant S., Bi Y.M., Zhu T., Rothstein S.J. (2009). SAUR39, a small auxin-up RNA gene, acts as a negative regulator of auxin synthesis and transport in rice. Plant Physiol..

[28] Roosjen M., Paque S., Weijers D. (2018). Auxin response factors: Output control in auxin biology. J. Exp. Bot..

[29] Buechel S., Leibfried A., To J.P.C., Zhao Z., Andersen S.U., Kieber J.J., Lohmann J.U. (2010). Role of A-type ARABIDOPSIS RESPONSE REGULATORS in meristem maintenance and regeneration. Eur. J. Cell Biol..

[30] Gordon-Kamm B., Sardesai N., Arling M., Lowe K., Hoerster G., Betts S., Jones T. (2019). Using morphogenic genes to improve recovery and regeneration of transgenic plants. Plant.

[31] Rocha D.I., Monte-Bello C.C., Aizza L.C.B., Dornelas M.C. (2016). A passion fruit putative ortholog of the SOMATIC EMBRYOGENESIS RECEPTOR KINASE1 gene is expressed throughout the in vitro de novo shoot organogenesis developmental program. Plant Cell Tissue Organ Cult..

[32] Xu C., Cao H.F., Zhang Q.Q., Wang H.Z., Xin W., Xu E.J., Zhang S.Q., Yu R.X., Ye D.X., Hu Y.X. (2018). Control of auxin-induced callus formation by bZIP59–LBD complex in Arabidopsis regeneration. Nat. Plants.

[33] Zheng Q., Zheng Y., Perry S.E. (2013). AGAMOUS-Like15 promotes somatic embryogenesis in Arabidopsis and soybean in part by the control of ethylene biosynthesis and response. Plant Physiol..

[34] Haecker A., Gross-Hardt R., Geiges B., Sarkar A., Breuninger H., Herrmann M., Laux T. (2004). Expression dynamics of WOX genes mark cell fate decisions during early embryonic patterning in Arabidopsis thaliana. Development.

[35] Palovaara J., Hallberg H., Stasolla C., Hakman I. (2010). Comparative expression pattern analysis of WUSCHEL-related homeobox 2 (WOX2) and WOX8/9 in developing seeds and somatic embryos of the gymnosperm *Picea abies*. New Phytol..

[36] Lu Y., Liu Z., Lyu M., Yuan Y., Wu B. (2019). Characterization of JsWOX1 and JsWOX4 during Callus and Root Induction in the Shrub Species *Jasminum sambac*. Plants.

[37] Guo H.H., Guo H.X., Zhang L., Fan Y.J., Wu J.F., Tang Z.M., Zhang Y., Fan Y.P., Zeng F.C. (2020). Dynamic transcriptome analysis reveals uncharacterized complex regulatory pathway underlying genotype-recalcitrant somatic embryogenesis transdifferentiation in Cotton. Genes.

[38] Guo J.R., Sun B.X., He H.R., Zhang Y.F., Tian H.Y., Wang B.S. (2021). Current understanding of bHLH transcription factors in plant abiotic stress tolerance. Int. J. Mol. Sci..

[39] Li W.X., Pang S.Y., Lu Z.G., Jin B. (2020). Function and mechanism of WRKY transcription factors in abiotic stress responses of plants. Plant.

[40] Zhao P.C., Hou S.L., Guo X.F., Jia J.T., Yang W.G., Liu Z.J., Chen S., Li X., Qi D., Liu G. (2019). A MYB-related transcription factor from sheepgrass, LcMYB2, promotes seed germination and root growth under drought stress. BMC Plant Biol..

[41] Iwase A., Mitsuda N., Koyama T., Hiratsu K., Kojima M., Arai T., Inoue Y., Seki M., Sakakibara H., Sugimoto K. (2011). The AP2/ERF transcription factor WIND1 controls cell dedifferentiation in Arabidopsis. Curr. Biol..

[42] An H., Zhang J., Xu F., Jiang S., Zhang X. (2020). Transcriptomic profiling and discovery of key genes involved in adventitious root formation from green cuttings of highbush blueberry (*Vaccinium corymbosum* L.). BMC Plant Biol..

[43] Deng L., Wu Y., Li J., OuYang K., Ding M., Zhang J., Li S.Q., Lin M.F., Chen H.B., Hu X.S. (2006). Screening Reliable Reference Genes for RT-qPCR Analysis of Gene Expression in Moringa oleifera. PLoS ONE.

